# Discrimination of Hard Ticks by Polymerase Chain Reaction–Restriction Fragment Length Polymorphism (PCR-RFLP)

**DOI:** 10.3390/ijms27010285

**Published:** 2025-12-26

**Authors:** Nandhini Perumalsamy, Rohit Sharma, Ayyanar Elango, Ananganallur Nagarajan Shriram, Manju Rahi

**Affiliations:** 1 Indian Council of Medical Research—Vector Control Research Centre (ICMR-VCRC), Puducherry 605006, India; pnandhini015@gmail.com (N.P.); elangoar@yahoo.co.in (A.E.); drmanjurahi@gmail.com (M.R.); 2 Pondicherry University, Puducherry 605014, India

**Keywords:** hard ticks, genetic marker, ITS-2 genetic marker, PCR-RFLP

## Abstract

Hard ticks are important vectors for several human and zoonotic pathogens, transmitting diseases such as Crimean–Congo hemorrhagic fever, Lyme disease, Kyasanur forest disease, Powassan virus disease, Tick-borne encephalitis, Rickettsiosis, and Anaplasmosis. Morphological identification of ticks relies on taxonomic keys but is often challenging due to damaged, engorged, or immature specimens and requires expertise. Molecular taxonomy can be a supplement to species identification and usually requires nucleotide sequencing of the genetic markers. PCR-RFLP is an important tool for tick identification and can be supplemented to the classical taxonomy. The current study focused on the morphological identification of important hard tick vectors from India, their phylogenetic positioning, and developing a PCR-RFLP based diagnostic tool for easy identification of hard tick vectors. The primer sets were designed to amplify the ITS-2 region from important tick vectors causing human and zoonotic diseases in India. These ticks were morphologically identified with taxonomical keys, and the extracted genomic DNA were used for ITS-2 based PCR amplification. The nucleotide sequences from each vector were used for their phylogenetic positioning. We obtained variable sizes of ITS-2 amplicons from each species and utilized the sequence for RFLP assays design. We have successfully shown PCR-RFLP based assays with two different restriction enzymes (*Hae* III & *Rsa* I) with specific restriction sites on the amplified regions. The PCR-RFLP tool showed different DNA fragment patterns on the agarose gel, specific for each hard tick vector. This study presents the phylogenetic positioning of Indian tick vectors and demonstrates the development and applicability of a molecular tool for their identification.

## 1. Introduction

Ticks are a common ectoparasite of amphibians, reptiles, birds, and mammals, which also parasitize domestic and pet animals [1]. They are the second most important arthropod vectors after mosquitoes, transmitting diseases to humans and animals [2]. Ticks can acquire, maintain, and transmit a broader range of pathogens, including viruses, bacteria, and protozoa, than any other blood-feeding arthropods [3,4]. Nearly 900 species of ticks are well-known. These species are divided into three families: Ixodidae/hard ticks (700 species), Argasidae/soft ticks (200 species), and Nuttaliellidae (monotypic family) [5,6]. Among these, hard ticks are the most significant in the medical and veterinary fields. *Haemaphysalis*, *Rhipicephalus*, *Hyalomma*, *Dermacentor*, *Ixodes*, and *Amblyomma* are widely distributed and serve as vectors for various diseases, predominantly affecting domestic animals, though some species also parasitize humans [2,7,8,9,10].

Ticks form a highly localized population, and the pathogens they transmit may vary geographically due to environmental and climatic factors [11,12]. The distribution of ticks and their role in disease transmission are influenced by habitat expansion, climate change, and host availability, with global concerns rising due to the geographical and range expansion and increasing tick-borne diseases (TBDs) [13,14]. Several hard tick species are responsible for transmitting pathogens to humans and animals. *Hyalomma anatolicum anatolicm* is the primary vector for Crimean–Congo Hemorrhagic Fever (CCHF), and other *Hyalomma* spp. are also involved in CCHF transmission [10]. *Haemaphysalis spinigera* is the main vector for Kyasanur Forest Disease (KFD) [15,16]. Indian tick typhus and other rickettsial pathogens are transmitted by *Rhipicephalus sanguineus* [2,17]. *Rhipicephalus (Boophilus) microplus* is involved in the transmission of babesiosis, theileriosis, and anaplasmosis throughout the world [18]. Ticks in the *Amblyomma* genus are known vectors for multiple diseases like rocky mountain spotted fever, tularemia, ehrlichiosis, KFD, and other rickettsial pathogen transmission [19,20].

Morphological identification of these tick vectors relies on determining the shape, size, and color of ticks using taxonomic keys. However, morphological identification has limitations, requiring expertise and difficulties with immature, damaged, or engorged specimens, as well as closely related species [21,22]. Phylogenetic markers address these challenges, supporting species identification and population genetic studies [21,23]. Phylogenetic studies based on the molecular marker can allow us to understand the genetic structure, hybrid vectors, and genetic factors associated with acaricide resistance [2,24,25]. Mitochondrial and ribosomal genes like COI, 16S rRNA, 12S rRNA, ITS-1 (Internal Transcribed Spacer-1), and ITS-2 (Internal Transcribed Spacer-2) are the common markers for species identification and phylogenetic analysis [26,27,28]. The ITS-2 non-coding region, flanked by the conserved rDNA genes (5.8S, and 28S), evolves rapidly due to minimal selection pressure, allowing it to accumulate substitutions that distinguish between closely related species. Primers targeting the conserved genes flanking ITS-2 enable effective amplification across diverse genera of hard ticks, making it a powerful tool for tick species identification [28,29]. Due to inter-species variations in ITS-2 nucleotide sequences resulting in variable PCR amplicon size, this is a valuable marker for developing an identification tool for tick vectors [29,30].

PCR-RFLP (Polymerase Chain Reaction-Restriction Fragment Length Polymorphism) is one such molecular technique which is rapid, cost-effective, and accurate in the identification of arthropod vectors [31]. Previous studies showed that the ITS-2 region-based PCR-RFLP had the best resolution among different species of ticks [32,33]. RFLP differentiates species by analyzing DNA band patterns generated by restriction enzymes. Variations in cleavage sites produce fragments of different lengths, allowing species and even strains to be distinguished based on their unique fragment profiles [34]. This method integrates PCR with RFLP, offering a simple, specific, and sensitive tool for tick identification. For instance, species within the *Rhipicephalus (Boophilus)* genus *R. microplus*, *R. geigyi*, *R. decoloratus*, and *R. annulatus* are distinguished using PCR-RFLP targeting the ITS-2 region, with restriction carried out by the enzyme *Msp*I [32]. Similarly, *Rhipicephalus appendiculatus* and *Rhipicephalus zambeziensis* are differentiated as distinct species using PCR-RFLP, where the ITS-2 genetic marker was restricted by the enzyme *Bau*I [33].

In this study, tick vectors were morphologically identified via dichotomous keys and then subjected to DNA extraction and molecular characterization using the ITS-2 genetic marker. Furthermore, ITS-2 sequences obtained from these tick vectors were utilized to design a PCR-RFLP tool which enabled accurate species identification of these vectors.

## 2. Results

### 2.1. Morphological Characterization

**Figure 1:**
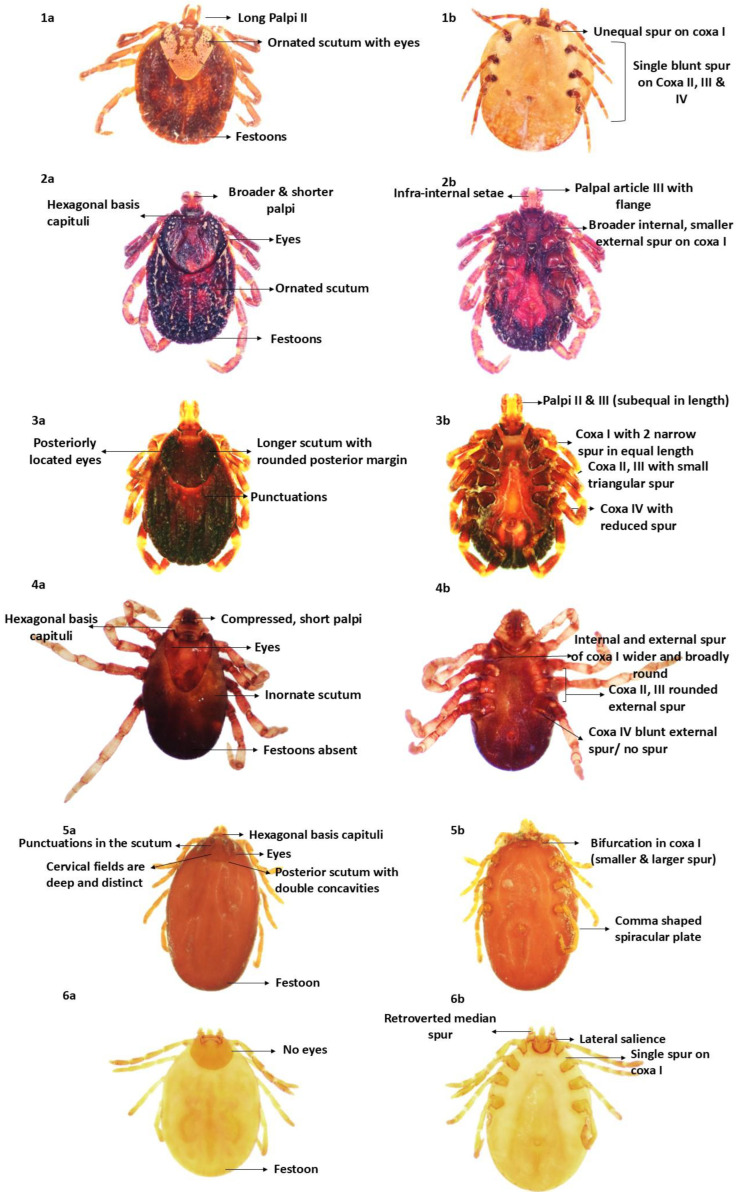
Morphology of the female hard ticks: (**1a**) (dorsal) and (**1b**) (ventral) view of *Amblyomma integrum*, (**2a**) (dorsal) and (**2b**) (ventral) view of *Nosomma monstrosum*, (**3a**) (dorsal) and (**3b**) (ventral) view of *Hyalomma anatolicum anatolicum*, (**4a**) (dorsal) and (**4b**) (ventral) view of *Rhipicephalus (Boophilus) microplus*, (**5a**) (dorsal) and (**5b**) (ventral) view of *Rhipicephalus sanguineus*, and (**6a**) (dorsal) and (**6b**) (ventral) view of *Haemaphysalis spinigera*.

Ticks were identified based on key morphological characteristics, including palpi structure, basis capitulum shape, body morphology, coloration, ornamentation, spiracle shape, adanal plate, eye presence, and festoon patterns [21,35]. The following species were identified based on established taxonomic descriptions (Figure 1) [36,37,38,39,40,41,42,43,44,45,46].

### 2.2. Primer Designing and PCR Amplification of ITS-2

**Figure 2:**
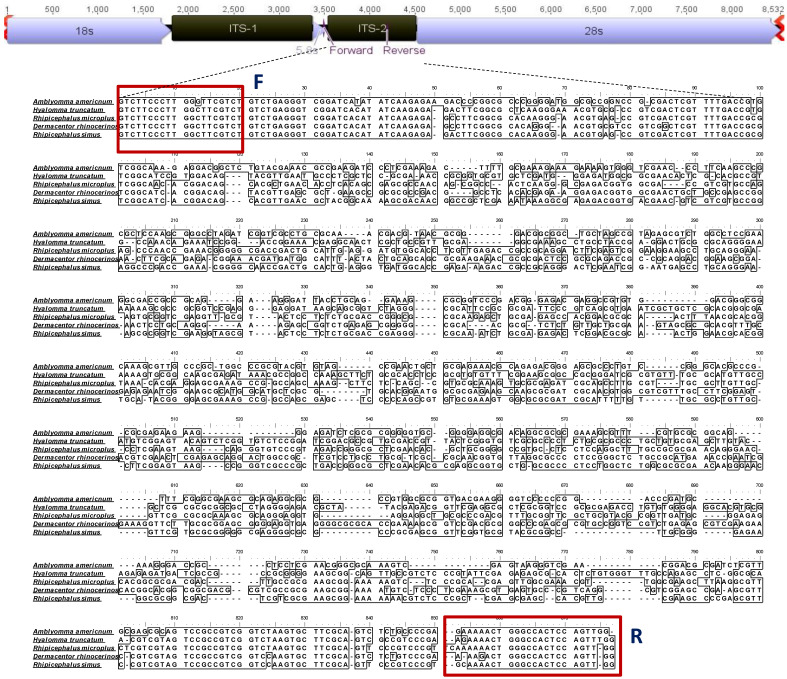
The multiple sequence alignment showing conserved regions of different hard tick species collected from NCBI and used for primer designing. This figure demonstrated the annotation of the nuclear fragment variation and gaps in the target fragment among different tick vectors from different genera.

The ribosomal spacer ITS-2 region was targeted for this study. The ITS-2 sequences used for primer designing were collected from NCBI, showing significant variations in the length; however, the used primer binding sites were conserved (Figure 2 and Table 1). We designed a degenerated primer set, which can produce amplicons from different tick vectors.

**Figure 3:**
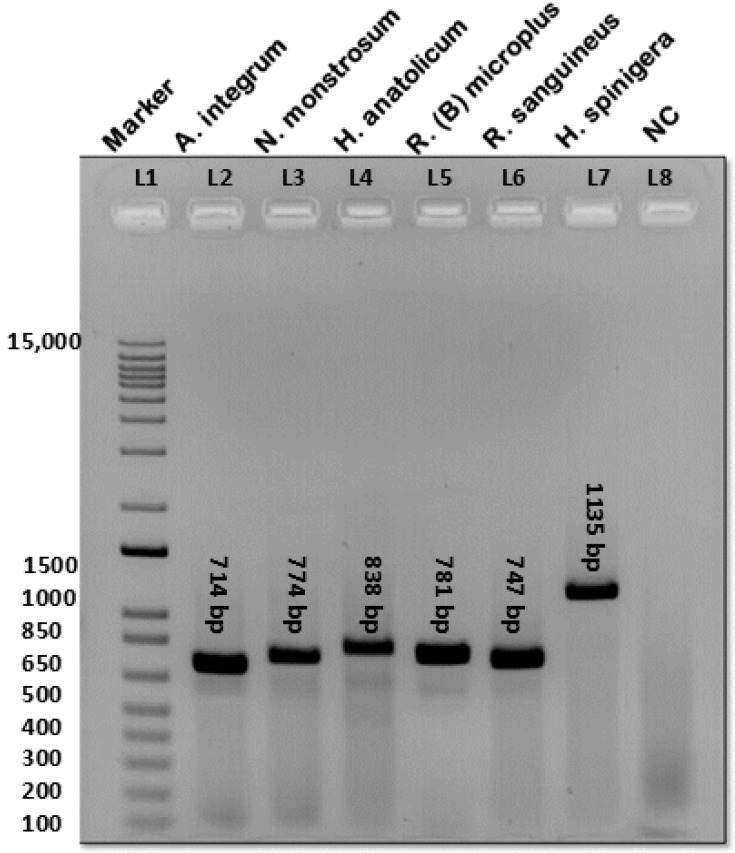
Amplification of ITS-2 fragment by using the designed primers. **L1**—Marker (1 kb plus), **L2**—*A. integrum* (714 bp), **L3**—*N. monstrosum* (774 bp), **L4**—*H. anatolium anatolicum* (838 bp), **L5**—*R. (B) microplus* (781 bp), **L6**—*R. sanguineus* (747 bp), **L7**—*H. spinigera* (~1200 bp), and **L8**—NC (negative control).

Morphologically identified tick samples were utilized for DNA extraction (Table S1). The extracted DNA from the individual tick vectors was utilized for the amplification of the ITS-2 region by using the designed primer. The amplified products were run on agarose gel electrophoresis, and the bands were confirmed for different ticks (Figure 3). We designed a degenerated primer set to produce amplicons (~700–1200 bp) from six tick vectors found in India (Figure 3). The PCR products were then purified, and Sanger sequenced resulting in different nucleotide lengths.

### 2.3. Sequence Analysis and Phylogenetic Positioning

**Figure 4:**
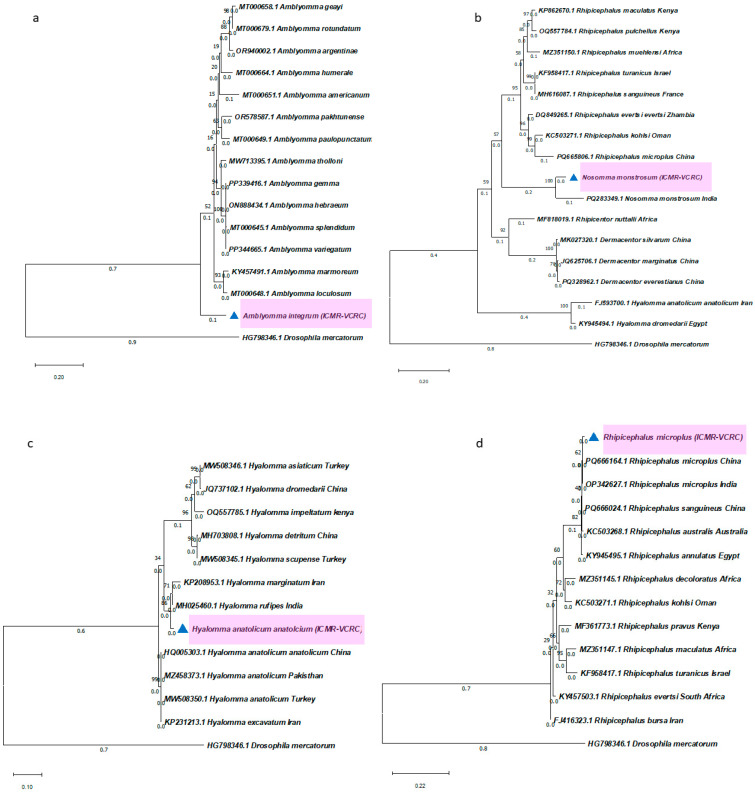

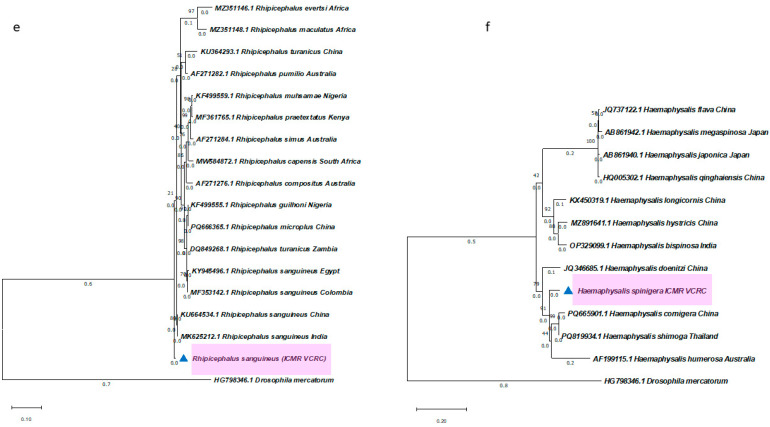
Phylogenetic positioning of ticks using the ITS-2 fragment. Phylogenetic trees were constructed using the Maximum Likelihood method (ML) with the Tamura–Nei model (1000 replicates) in MEGA 11. (**a**) *A. integrum*, (**b**) *N. monstrosum*, (**c**) *H. anatolicum anatolicum*, (**d**) *R. (B) microplus*, (**e**) *R. sanguineus*, and (**f**) *H. spinigera*. The color (pink) and the triangle (blue) indicate tick species characterized in this study.

Sequences obtained from the amplified PCR products were blasted in the NCBI platform, and the species were confirmed with the closest nucleotide identities. Sequences of all six ticks from different genera of the hard tick family were used for the phylogenetic tree construction. The phylogenetic tree was constructed with other closely related species retrieved from the NCBI database. The NCBI blast of *A. integrum* sequence was matched with other *Amblyomma* ticks from Africa and America. However, the phylogenetic tree showed a 10% genetic difference between *A. integrum* and other African and American *Amblyomma* ticks. It showed that *A. integrum* is genetically diverged from other *Amblyomma* ticks from other continents. The *N. monstrosum* blast result showed close alignment with *N. monstrosum* from India and other *Rhipicephalus* ticks. *N. monstrosum* is a monotypic tick, grouped with *N. monstrosum* in the same clade and grouped with *Rhipicephalus* ticks. It showed a distance from *Dermacentor* and *Hyalomma* ticks. *H anatolicum anatolicum*, *R. (B) microplus*, and *R. sanguineus* exhibit closeness with the species of the same genus from different parts of the world. *R. sanguineus* from Asia grouped, and the same species from other places were separated from the Asian group. *H. spinigera* is grouped with the other *Haemaphysalis* spp. from China and Thailand in the same clade, and it is closer to *Haemaphysalis cornigera* and *Haemaphysalis shimoga* (Figure 4).

### 2.4. In Silico Analysis of PCR-RFLP of Important Hard Tick Vectors

**Figure 5:**
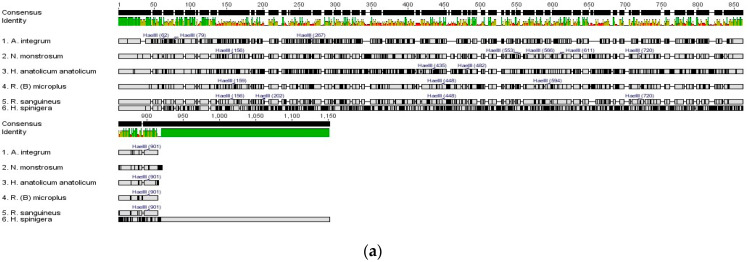

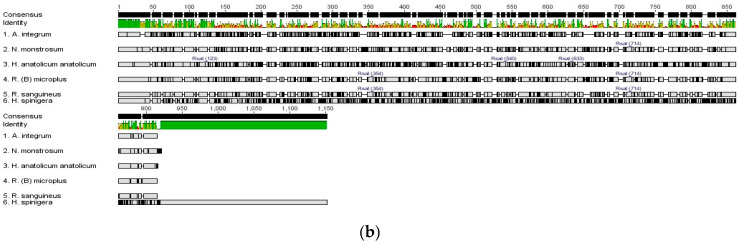
Restriction mapping of ITS-2 fragments from Indian tick vectors. (**a**) *Hae* III-based restriction digestion mapping. (**b**) *Rsa* I-based restriction digestion mapping.

The obtained ITS-2 sequence was mapped for unique restriction sites for commonly used enzymes. Finally, *Hae* III and *Rsa* I were selected for their diagnostic abilities. Both restriction enzymes could generate specific fragments of the PCR-amplified ITS-2 from different tick vectors. ITS-2 fragments of *A. integrum* (~720 bp) *and H. spinigera* (~1200 bp) are not digested by the *Rsa* I enzyme. But these species were differentiated by the variation in the length of the amplified product. These restriction fragments were unique due to the frequency and distance of the restriction sites, and the restriction sites for both enzymes in different tick vectors were mapped (Figure 5). Virtual gel was generated for each species using Geneious software version 9.0.5 (Figure S1). Moreover, for the other two common tick vectors from India, *Haemaphysalis bispinosa* and *Haemaphysalis intermedia*, ITS-2 sequences were collected from NCBI, and primer sites were annotated. The restriction map and virtual gel of the *Hae* III and *Rsa* I are shown in Figures S2 and S3.

### 2.5. PCR-RFLP Using Hae III and Rsa I

**Figure 6:**
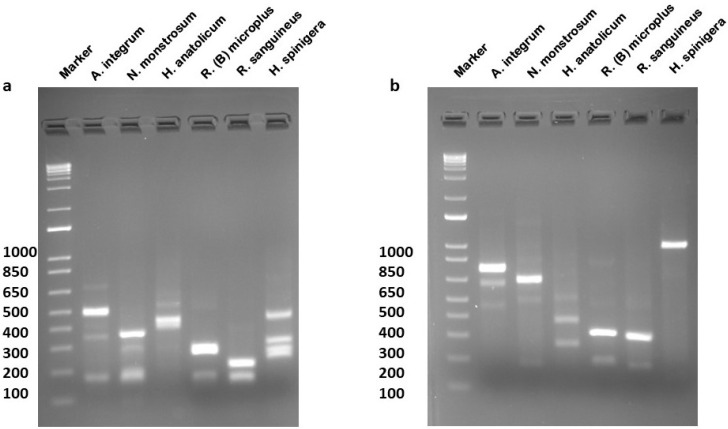
UV visualization of agarose gel electrophoresis of digested products of different tick vectors. (**a**) Restriction digestion of *Hae* III, (**b**) restriction digestion of *Rsa* I and Marker1kb plus marker.

PCR amplification was performed for each species, and the bands were confirmed as mentioned above (Figure 3). After restriction digestion, the prepared gel showed a distinct pattern of bands specific to each species of hard tick vectors (Table 2, Figure 6) and uncropped gel (Figure S5). The fragment length was compared with the virtual gel as mentioned (Figures S1 and S6). Several independent experiments were performed to determine the reproducibility of the developed PCR-RFLP assay (Figures S4 and S8). *Hae* III has more restriction sites compared to *Rsa* I. *A. integrum*, and *H. spinigera* was not digested by *Rsa* I. Due to a larger number of restriction sites for *Hae* III, the amplicons were digested into smaller bands in all species. *Rsa* I showed a low number of restriction sites, and the digested bands were larger than *Hae* III. The lower bands less than 100bp were not resolved, therefore not shown (Figure 6).

### 2.6. In Silico Analysis of PCR for Global Tick Vectors

**Figure 7:**
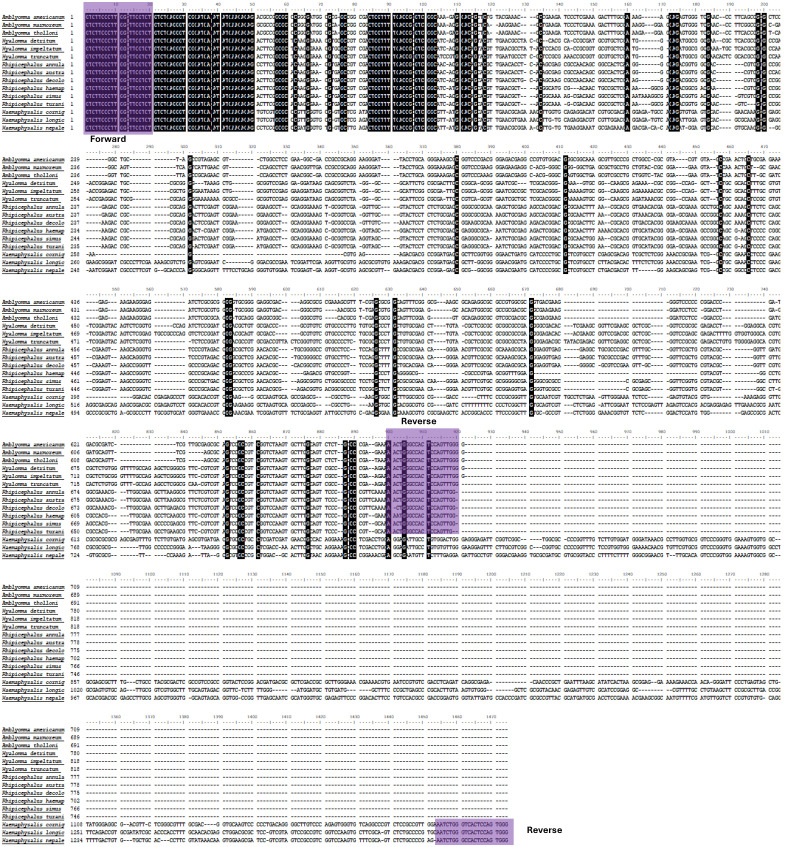
Multiple sequence alignment from ITS-2 region of various tick vectors, showing conserved primer binding sites. The black shade regions are the conserved region among different tick vectors.

**Figure 8:**
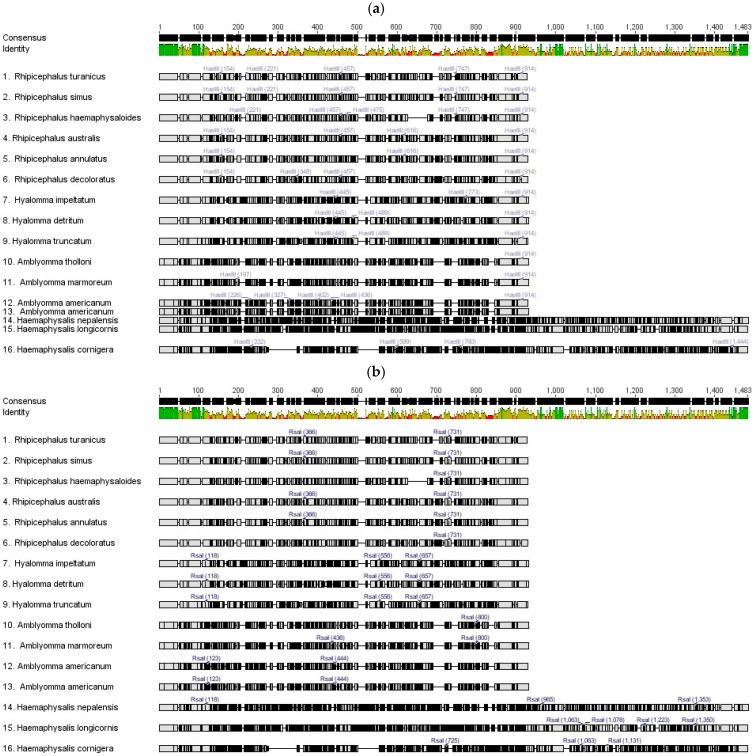
Restriction mapping of ITS-2 fragments from different tick vectors around the world. (**a**) *Hae* III based restriction digestion mapping. (**b**) *Rsa* I based restriction digestion mapping.

**Figure 9:**
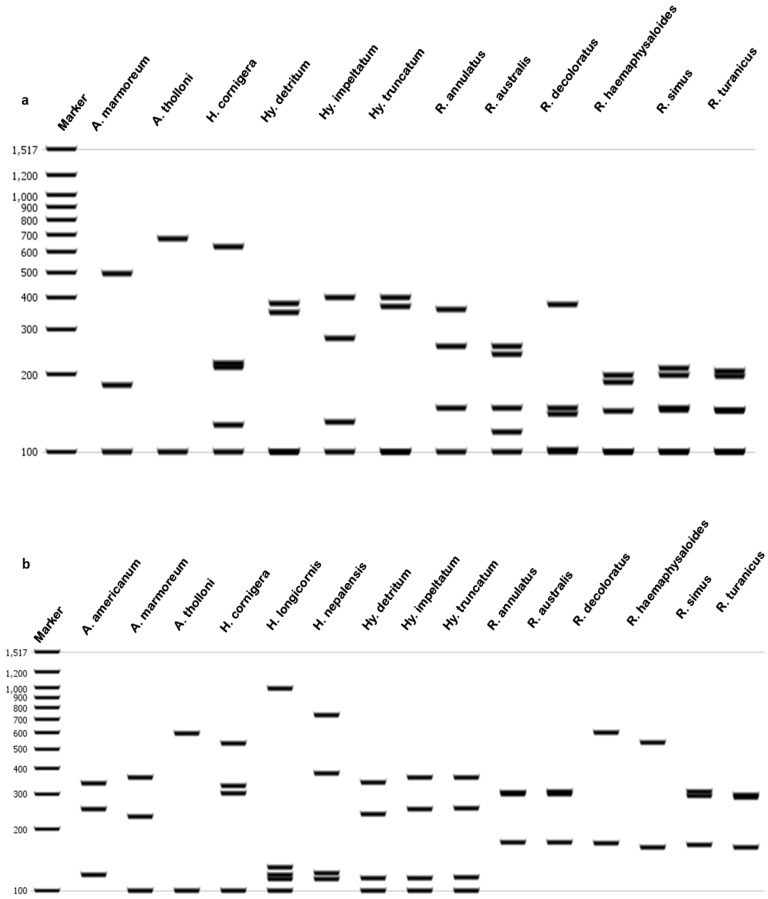
Virtual gel of other tick vectors from different genera. (**a**) Digestion with *Hae* III restriction enzyme (*A. americanum* (709 bp), *H. longicornis* (1372 bp), and *H. nepalensis* (1349 bp) no restriction sites). (**b**) Digestion with *Rsa I* restriction enzyme.

Moreover, in the in silico analysis, these restriction enzymes were used for other global tick vectors. For that, the available sequences of ITS-2 from the NCBI platform were collected, and the primer sites were annotated (Figure 7 and Figure S7). For three individual ticks of each genus, ITS-2 sequences of different genera of hard ticks and in silico digestion was performed by *Hae* III and *Rsa* I, and restriction mapping was constructed using the Geneious software (Figure 8). The virtual gel was finally produced for each tick species (Figure 9). *Amblyomma americanum*, *Amblyomma marmoreum*, and *Amblyomma tholloni* are the ticks from the *Amblyomma* genus that were clearly differentiated by the digestion of both enzymes. The *Haemaphysalis* ticks (*Haemaphysalis cornigera*, *Haemaphysalis longicornis*, and *Haemaphysalis nepalensis*) were well differentiated with *Rsa* I and *H. longcornis* (1372 bp) and *H. nepalensis* (1349 bp) did not have a restriction site for *Hae* III. However, these species showed slight length variation without digestion.

*Hyalomma detritum*, *Hyalomma impeltatum*, and *Hyalomma truncatum* were the species from the *Hyalomma* genus screened for restriction digestion with *Hae* III and *Rsa* I. *Hae* III showed well differentiated band patterns for each tick, and *Rsa* I showed more or less similar band patterns. *Rhipicephalus annulatus*, *Rhipicephalus australis*, and *Rhipicephalus decoloratus* are closely related tick species, which showed unique patterns for both *Hae* III and *Rsa* I. *Hae* III showed better variations among these species. *Rsa* I showed the differentiation between *Rhipicephalus haemaphysaloides*, *Rhipicephalus simus*, and *Rhipicephalus turanicus*. Finally, both the enzymes are capable of differentiating the hard ticks between genus and within genus as well.

## 3. Discussion

The molecular method for tick identification can be a supplementary tool for classical taxonomy and enhance the accuracy [20]. The abundance of tick vectors is usually an indicator of the risk for transmission of associated pathogens in the locality, indicating the importance of tick surveillance in different geographies [47]. Identification of ticks is challenging and requires specialized skills, and it is difficult to identify immature and damaged ticks by morphology even by an expert [48]. The morphological differences between some of the genera are low, especially in female ticks [49]. Molecular level identification of ticks is needed to exclude all these gaps, and it supports the classical taxonomy. In India, molecular taxonomy is not well documented, due to the absence of molecular tools for the identification of local tick species. Therefore, to develop such tools requires the accurate morphological identification and molecular characterization of these ticks.

PCR-RFLP is a cost-effective molecular tool utilized for species differentiation among genera across plants and animals, including important parasites and vectors [34]. The PCR amplicons of the gene of interest, digested with restriction enzymes can differentiate species by DNA band patterns analyzed on agarose gel. Different genetic markers like ITS-1, ITS-2, HSP60, COI, 16S r DNA, and 18S rDNA have proven useful for PCR-RFLP to differentiate various parasites and vectors [50,51,52,53,54,55,56]. The ITS-2 region was previously used for developing RFLP tools and employed for differentiating various species of trematodes and nematodes including *Fasciola* spp., *Trichostrongylus* spp., *Cylicocyclus* spp., *Anisakis simplex*, *Trichuris* spp., and *Strongylus* spp. [57,58,59,60,61,62,63,64,65,66]. The ITS-based RFLP has been widely utilized for the molecular identification of fungal and protozoan infections like *Candida* spp., *Leishmania* spp., *Crithidia* spp., and *Cryptosporidium* spp. [64,65,66,67]. There are several studies in which ITS2-RFLP was used as a powerful tool for species differentiation of malaria vectors [31,68,69,70].

In this study, we have developed a PCR-RFLP assay for identifying important Indian hard tick vectors. The classical taxonomic keys were used to morphologically identify these tick vectors, and molecular phylogenetic analysis was performed to confirm the genera/species. The developed assay involved amplification of ITS-2 region conserved at the primer binding site with variation in the length of the fragment. A single designed degenerate primer set was utilized for the PCR amplification of ITS-2 with optimized PCR conditions. These amplified regions of the ITS-2 fragment from different tick species were able to differentiate them based on different lengths of amplicons. Furthermore, restriction enzymes like *Rsa* I and *Hae* III showed distinct fragment patterns specific for each tick species. The different restriction fragment patterns obtained for each species were analyzed to develop a discrimination method using PCR-RFLP analysis without the need for sequencing. Furthermore, our PCR-RFLP method showed that a higher sensitivity and specificity to six tick species found in India can be determined within a short time. The six species used in this study were successfully distinguished using two different restriction enzymes (*Hae* III and *Rsa* I) with a designed degenerate ribosomal primer set.

In a previous study, ITS-2 RFLP has been used for differentiation of tick species from *Rhipicephalus (Boophilus)* genus, including *R. microplus*, *R. geigyi*, *R. decoloratus*, and *R. annulatus* using the *Msp*I restriction enzyme [32]. In other studies, ITS-2 RFLPs were used to differentiate the species of the *Rhipicephalus* genus, *R. appendiculatus* and *R. zambeziensis*, using *Bau* I, and *H. bispinosa* and *Boophilus microplus,* using *Hind* III [33,71]. Furthermore, PCR-RFLP with COI and 16S rRNA was demonstrated to differentiate *Haemaphysalis* ticks [72].

The developed PCR-RFLP was validated for important tick vectors belonging to six genera including *A. integrum*, *N. monstrosum*, *H. anatolicum anatolicum*, *R. (B) microplus*, *R. sanguineus*, and *H. spinigera*. The result indicated that the PCR-RFLP assay has the potential to discriminate these vectors based on the restricted band patterns of ITS-2. This PCR-RFLP tool can be used for identifying tick vectors and may be a supplement to classical taxonomy. Furthermore, we demonstrated that this PCR-RFLP assay may be suitable for discriminating global tick vectors. The in silico analysis showed conserved primer binding sites in the available ITS-2 sequences obtained from global tick vectors. The restriction sites mapping for *Rsa* I and *Hae* III predicted differential band patterns in virtual gel and suggested the utility of the developed PCR-RFLP in discriminating tick vectors from different parts of the world.

## 4. Materials and Methods

### 4.1. Study Area and Tick Collection

**Figure 10:**
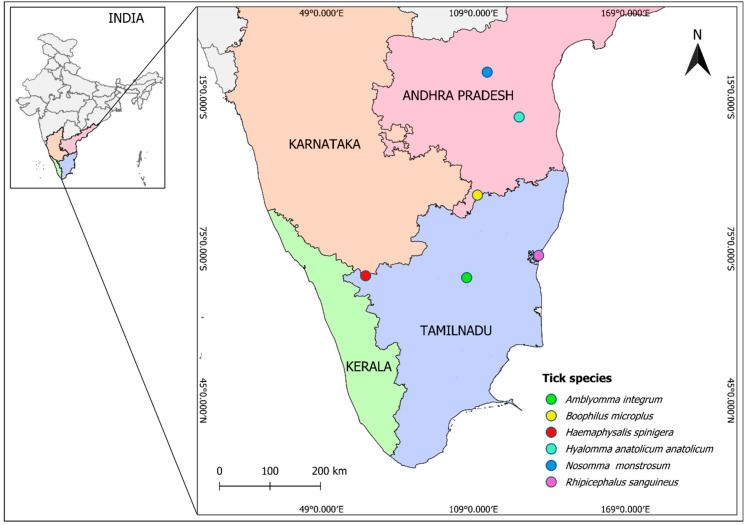
Map displaying the sites where the tick vectors collected from domestic animals in India. The collected ticks from these regions were initially characterized (morphology and molecular), and the obtained sequences were utilized for restriction mapping. The map was generated by using QGIS Desktop 3.34.13 software.

Ticks were collected from domestic animals (*Bos taurus*, *Bubalus bubalis*, *Capra aegagrus hircus*, *Ovis aries*, and *Canis lupus familiaris*), as a part of tick surveys conducted in southern parts of India, between 2022 and 2023. The tick collection sites included the states of Tamil Nadu (Keeripatti (11°32′19″ N 78°29′28″ E) and Masinangudi (11°34′26″ N 76°38′58″ E)), Andhra Pradesh (Obulapuram Thanda (15°17′11″ N 78°51′53″ E), Thopugunta (14°28′06″ N 79°26′51″ E), and Thimmaigaripalle (13°02′41″ N 78°41′13″ E)), and Puducherry (11°56′35″ N 79°48′09″ E) (Figure 10). Ticks were carefully removed from hosts using sterile forceps and preserved in 80% ethanol for subsequent analysis. Overall, approximately 50 ticks (females and nymphs) were used for this study from six different genera. *A. integrum*, *N. monstrosum*, *H. anatolicum anatolcium*, *R. (B) microplus*, *R. sanguineus*, and *H. spinigera* are the six tick species identified by using the developed PCR-RFLP. To ensure optimal preservation, the specimens were stored in a deep freezer (−40 °C) until further processing.

### 4.2. Morphological Characterization

Morphological identification was conducted using a stereo zoom microscope (Weswox SZM-102, The Western Electric and Scientific Works (Weswox), Ambala, Haryana, India). Identification was based on established dichotomous keys, considering morphological characteristic such as body shape, presence of festoons, eye morphology, palpal length, spur arrangement, basis capitulum shape, adanal plate presence, and dental formula [36,37,38,39,40,41,42,43,44,45,46].

### 4.3. Molecular Characterization

#### 4.3.1. DNA Extraction

Total genomic DNA was extracted from individual tick specimens (n = 60 ticks from six different genera) using the DNeasy Blood and Tissue Kit^®^ (Qiagen, Hilden, Germany), following the optimized protocol [20]. The DNA quality and concentration were assessed using a Thermo NanoDrop Lite UV–Visible Spectrophotometer^®^ (Waltham, MA, USA) before proceeding to downstream applications (Table S1).

#### 4.3.2. Primer Designing

Available sequences from different tick vectors from the NCBI database were used for MSA (Multiple Sequence Alignment) using BioEdit v7.2.5 software, and a common conserved region flanking a variable region was selected for the primer binding sites specific to various genera of hard ticks (Table S2). The designed primers were validated, synthesized, and subsequently used for PCR amplification (Figure 2) (Table 1). The GC content and primer dimers were checked with the IDT OligoAnalyzer™ Tool (https://www.idtdna.com/pages/tools/oligoanalyzer, accessed on 20 December 2025). The primers were expected to amplify ITS-2 fragments from all Indian tick vectors.

#### 4.3.3. PCR Amplification, Sequencing, and Phylogenetic Analysis

The designed primers were utilized to amplify the targeted ITS-2 fragment. Each PCR reaction included 2 µL of the DNA sample, with gene amplification performed using the Q5 Master Mix (New England Biolabs, Ipswich, MA, USA) and a Bio-Rad (Hercules, CA, USA) T100 Thermal Cycler^®^. The PCR protocol comprised an initial denaturation at 98 °C for 1 min, followed by 35 cycles of denaturation at 98 °C for 10 s, annealing at 59 °C for 15 s, and extension at 72 °C for 20 s. A final extension step was carried out at 72 °C for 5 min. The amplified PCR products were run on a 1.5% agarose gel and visualized under UV light using the VILBER Bioprint^®^ gel documentation system. The PCR products were purified using the PCR Clean-up Kit (Macherey-Nagel, Düren, Germany) and subsequently sequenced using the Sanger method. The resulting nucleotide sequences were identified as the ITS-2 region of hard ticks through an nBLAST search on the NCBI database. Multiple sequence alignments were performed using BioEdit v7.2.5 software (https://bioedit.software.informer.com/7.2/, accessed on 20 December 2025). Phylogenetic analysis was conducted using the Maximum Likelihood (ML) method with 1000 bootstrap replicates, applying the Tamura–Nei substitution model in MEGA 11 software.

#### 4.3.4. In Silico Analysis for PCR-RFLP for Important Tick Vectors

The obtained sequences of the ITS-2 fragment from Indian tick vectors were aligned together, the forward and reverse primers were annotated on the sequences, and length variations were confirmed. The individual sequences were screened with different restriction enzymes using Geneious version9.0.5 software, and common enzymes that restrict different positions for different species were selected. In this study, for the PCR-RFLP analysis of Indian tick vectors, *Hae* III and *Rsa* I restriction enzymes were selected. *Rsa* I recognizes and restricts the sequences at GT↓AC, and *Hae* III recognizes the sequences at GG↓CC and restricts it. The virtual gels were made for different species. Further, the ITS-2 sequences of other tick vectors distributed in the different parts of the world were collected from the NCBI, and the collected sequences were aligned using MSA with BioEdit v7.2.5 software. The forward and primer sites were annotated, and restriction mapping was performed with *Rsa* I and *Hae* III. The virtual gel was made for each tick vector.

#### 4.3.5. Development of PCR-RFLP (Restriction Fragment Length Polymorphism) Based Assay of ITS-2 for Indian Tick Vectors

PCR was performed by using an optimized protocol, and amplification was confirmed. The restriction enzymes *Rsa* I (Sisco Research Laboratories Pvt. Ltd.—97505, Mumbai, India) and *Hae* III (Promega R6175, Madison, WI, USA) were used for restriction. For the restriction, 25 µL of reaction mix was prepared, which includes 2.5 µL of 10× buffer, 0.5 µL of restriction enzyme, and 18.5 µL of nuclease-free water with 3.5 µL amplicon, and was mixed well. The reaction mix was left overnight at 37 °C. All restricted samples were mixed with gel loading dye and loaded to 2.5% agarose gel, and a 1kb^+^ DNA ladder (Invitrogen™ 1 Kb Plus DNA Ladder, Carlsbad, CA, USA) was added to the gel for fragment size determination. The gel was stained with safe stain (HiMedia-Hi-SYBr Safe Gel Stain (Kennett Square, PA, USA) (10,000× in DMSO) and visualized under UV light.

## 5. Conclusions

This study involves the molecular characterization of hard tick vectors co-existing in India. We have developed a PCR-RFLP-based molecular tool for the identification of these vectors, which can be crucial for tick taxonomy in India. This PCR-based method is easy, does not require sequencing for tick identification, and can be utilized for important tick vectors globally. As TBDs are public health threats in India, the development of such molecular tools, like PCR-RFLP for tick identification, is important, given that morphological identification requires special expertise in tick taxonomy and is not easily available. We have now validated the tool for identifying at least six different genera involved in the transmission of important diseases like KFD, CCHF, Indian tick typhus, and other rickettsial pathogens. However, intra-specific variations among these tick vectors should be analyzed for any potential variations on the restriction sites chosen for PCR-RFLP. Considering the low intraspecific variation in ITS-2, this developed PCR-RFLP tool cannot be used for strain determination and may not be useful for hybrid tick identification. In conclusion, the developed tool has a potential role in tick surveys for the risk assessment of re-emerging outbreaks of TBDs.

## Figures and Tables

**Table 1:** Primer sequences used to amplify the ITS-2 spacer from the tick vectors from different genera. The primer was used to amplify ITS-2 regions from Indian tick vectors.

Primer Name	Forward	Reverse	Product Size
ITS2	5′-GTCTTCCCTTGGCTTCGTCT-3′	5′-CCMACTGGAGTGRCCCAGWT-3′	~700–1200 bp

**Table 2:** The primers synthesized using available NCBI sequences were used to amplify the ribosomal internal transcribed spacer 2 (ITS2) region from adult tick vectors. The amplified regions were utilized for phylogenetic analysis and restriction fragment length polymorphism (RFLP) studies.

Tick Species	Hae III		Rsa I	
	Restriction Sites	Band Sizes	Restriction Sites	Band Sizes
*Amblyomma integrum*	4	466 bps, 164 bps, 54 bps 17 bps, and 13 bps	0	No restriction site
*Boophilus microplus*	4	258 bps, 240 bps, 152 bps,118 bps, and 13 bps	2	306 bps, 304 bps, and 171 bps
*Hyalomma anatolicum anatolicum*	3	405 bps, 379 bps, 40 bps, and 14 bps	3	365 bps, 263 bps, 120 bps, and 90 bps
*Nosomma monstrosum*	5	327 bps, 166 bps,145 bps, 86 bps, 41 bps, and 9 bps	1	602 bps and172 bps
*Rhipicephalus sanguineus*	5	205 bps,198 bps, 143 bps,145 bps, 43 bps, and 13 bps	2	295 bps, 288 bps, and 164 bps
*Haemaphysalis spinigera*	3	391 bps, 284 bps 243 bps, and 217 bps	0	No restriction site

## Data Availability

The original contributions presented in this study are included in the article/Supplementary Material. Further inquiries can be directed to the corresponding author.

## Supplementary Materials

The following supporting information can be downloaded at https://www.mdpi.com/article/10.3390/ijms27010285/s1.

## Abbreviations

TBDs	Tick-borne Diseases
PCR–RFLP	Polymerase Chain Reaction–Restriction Fragment Length Polymorphism
ITS-2	Internal Transcribed Spacer-2
CCHF	Crimean–Congo Hemorrhagic Fever
KFD	Kyasanur Forest Disease
NCBI	National Center for Biotechnology Information
MSA	Multiple Sequence Alignment
