# Supplementary material for: Discrimination of Hard Ticks by Polymerase Chain Reaction–Restriction Fragment Length Polymorphism (PCR-RFLP)
Source: Int J Mol Sci. 2025 Dec 26;27(1):285. doi: 10.3390/ijms27010285 (PMC12786177; doi:10.3390/ijms27010285)
Supplement: Supplementary file 1 [file ijms-27-00285-s001.zip › ijms-3971473-supplementary.pdf]

**Species**	Concentration (ng/ul)	Purity A (260/280)
Amblyomma integrum	77.1	1.89
Nosomma monstrosum	56.1	1.71
Hyalomma anatolicum	68.0	1.78
Rhipicephalus (Boophilus) microplus	296.2	2.1
Rhipicephalus sanguineus	46.3	1.99
Haemaphysalis spinigera	78.8	1.94

Table S1: Genomic DNA concentration and purity, extracted from individual tick vectors (extracted by using Qiagen blood and tissue kit).

**Name of the species**	**Gene Bank ID**
Amblyomma americanum	MT000651.1
Amblyomma marmoreum	KY457491.1
Amblyomma tholloni	MW713395.1
Haemaphysalis cornigera	PQ66590.1
Haemaphysalis nepalensis	ON660526.1
Haemaphysalis longicornis	KX450329.1
Hyalomma detritum	MH703808.1
Hyalomma impeltatum	OQ557785.1
Hyalomma truncatum	KY457496.1
Rhipicephalus annulatus	KY945495.1
Rhipicephalus australis	KC503268.1
Rhipicephalus decoloratus	MZ351145.1
Rhipicephalus haemahysaloides	JQ737126.1
Rhipicephalus simus	KY457508.1
Rhipicephalus turanicus	KF958417.1
Dermacentor rhinocerinus	KY457494.1
Rhipicephalus microplus	KY457506.1

Table S2: Gene bank ID of tick species retrieved from NCBI data bank for primer designing and Insilico analysis.

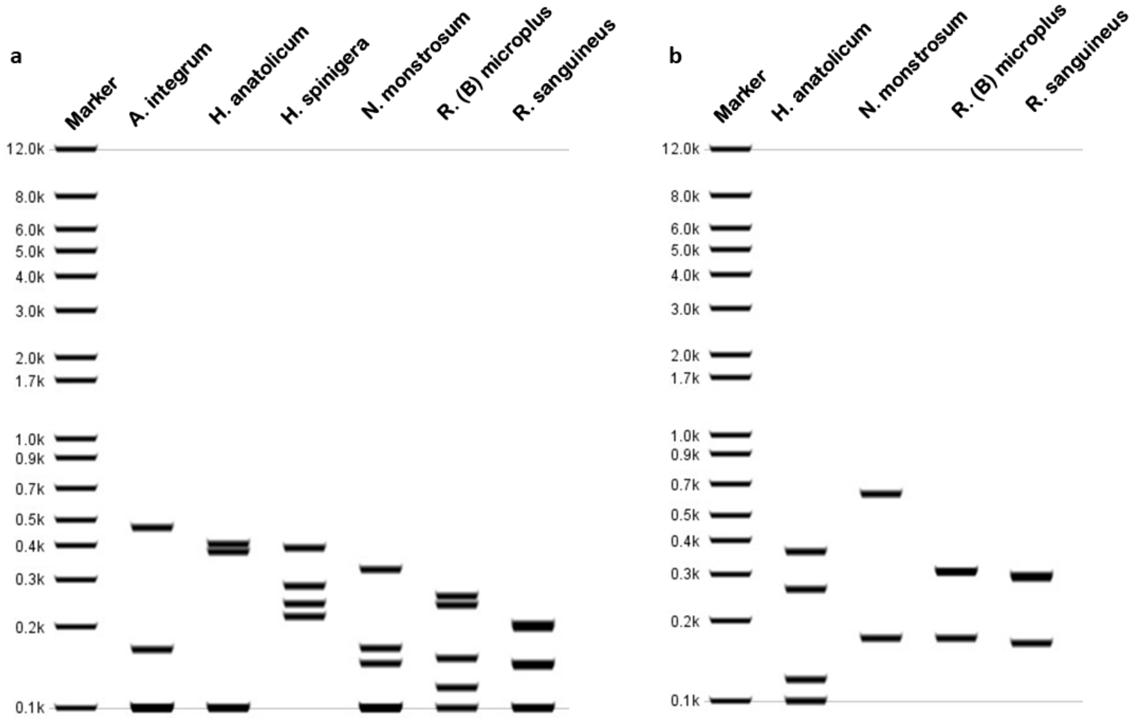

Figure S1: Virtual gel of Indian tick vectors-based on *Hae* III and *Rsa* I restriction digestion. **a)** Profile of *Hae* III based restriction digestion. **b)** Profile of *Rsa* I based restriction digestion (*H. spinigera* (~1200bps) and *A. integrum* (~ 720 bps) showed no restriction site for *Rsa* I)

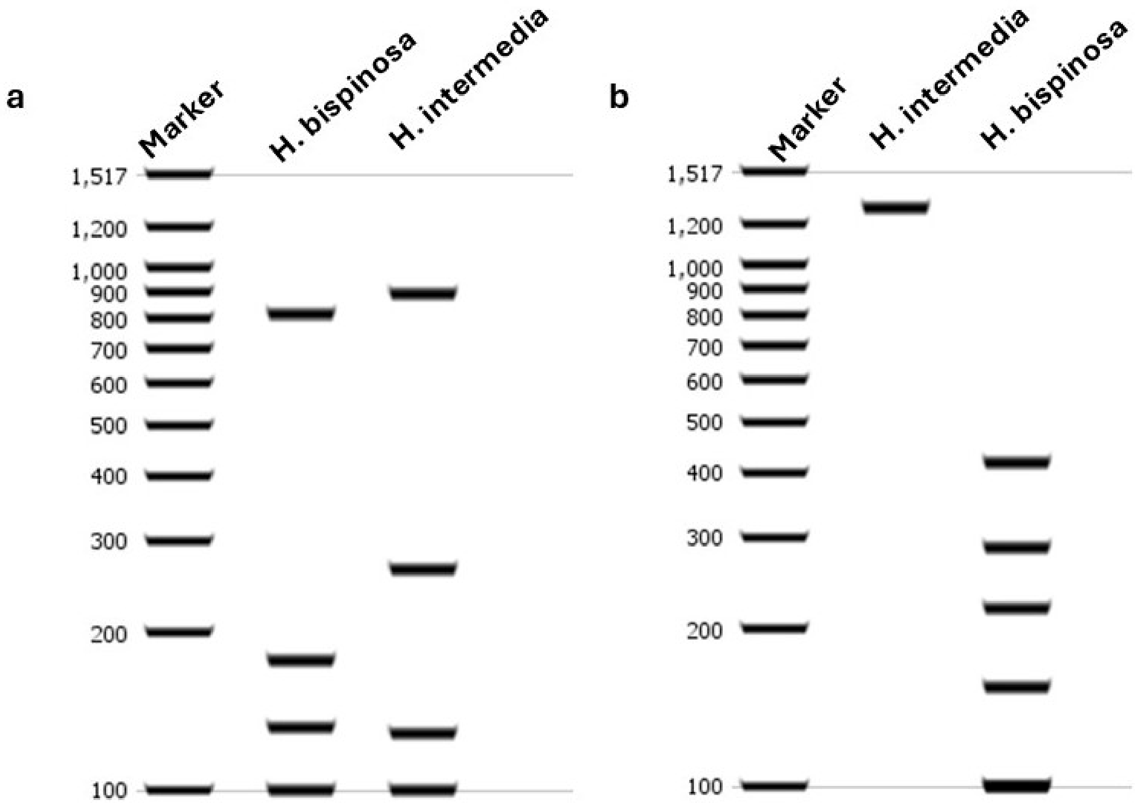

Figure S2: a) Restriction digestion with *Rsa* I and b) Restriction digestion with *Hae* III.

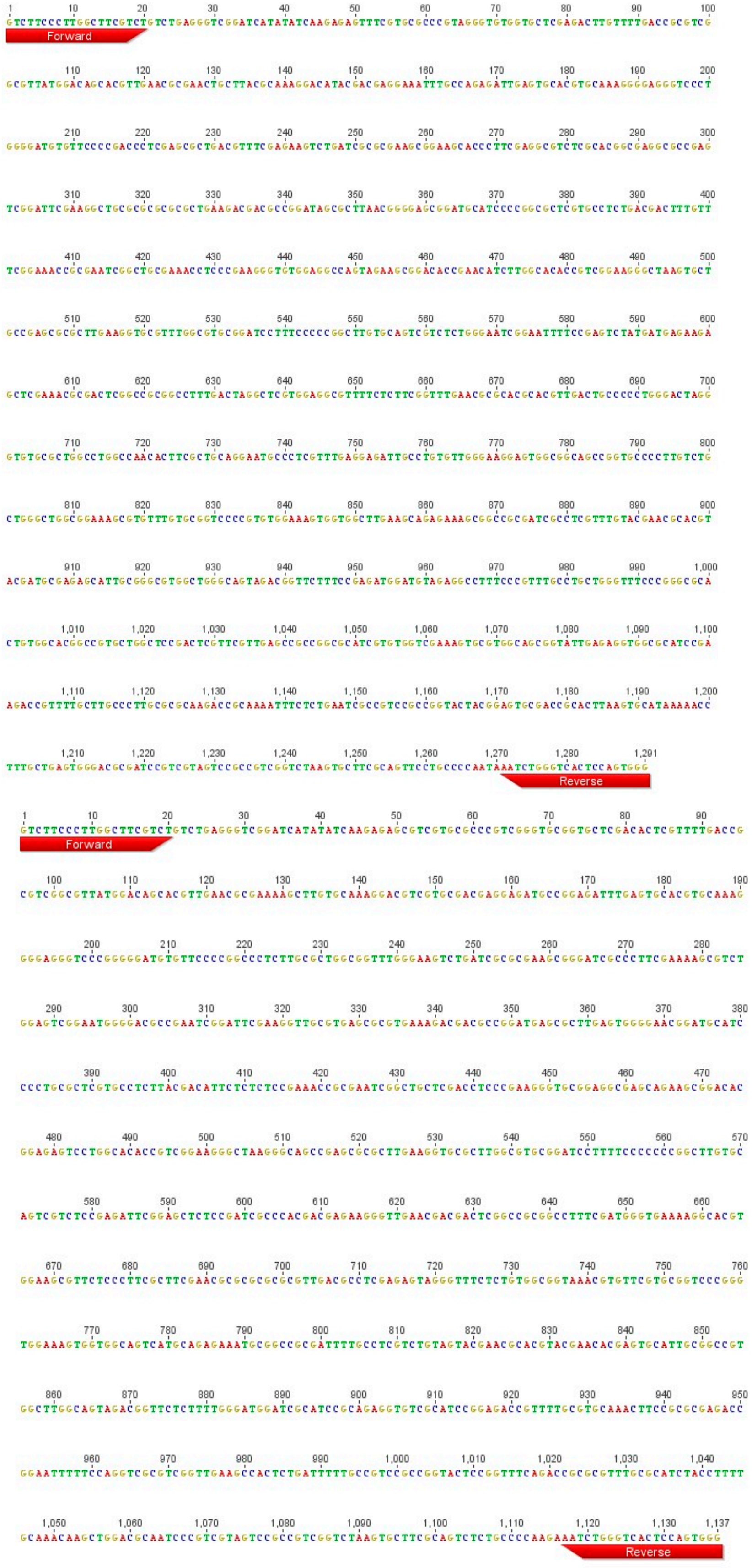

Figure S3: Primer annotations of *H. intermedia* (>PV740275.1), and *H. bispinosa* (>KC853416.1).

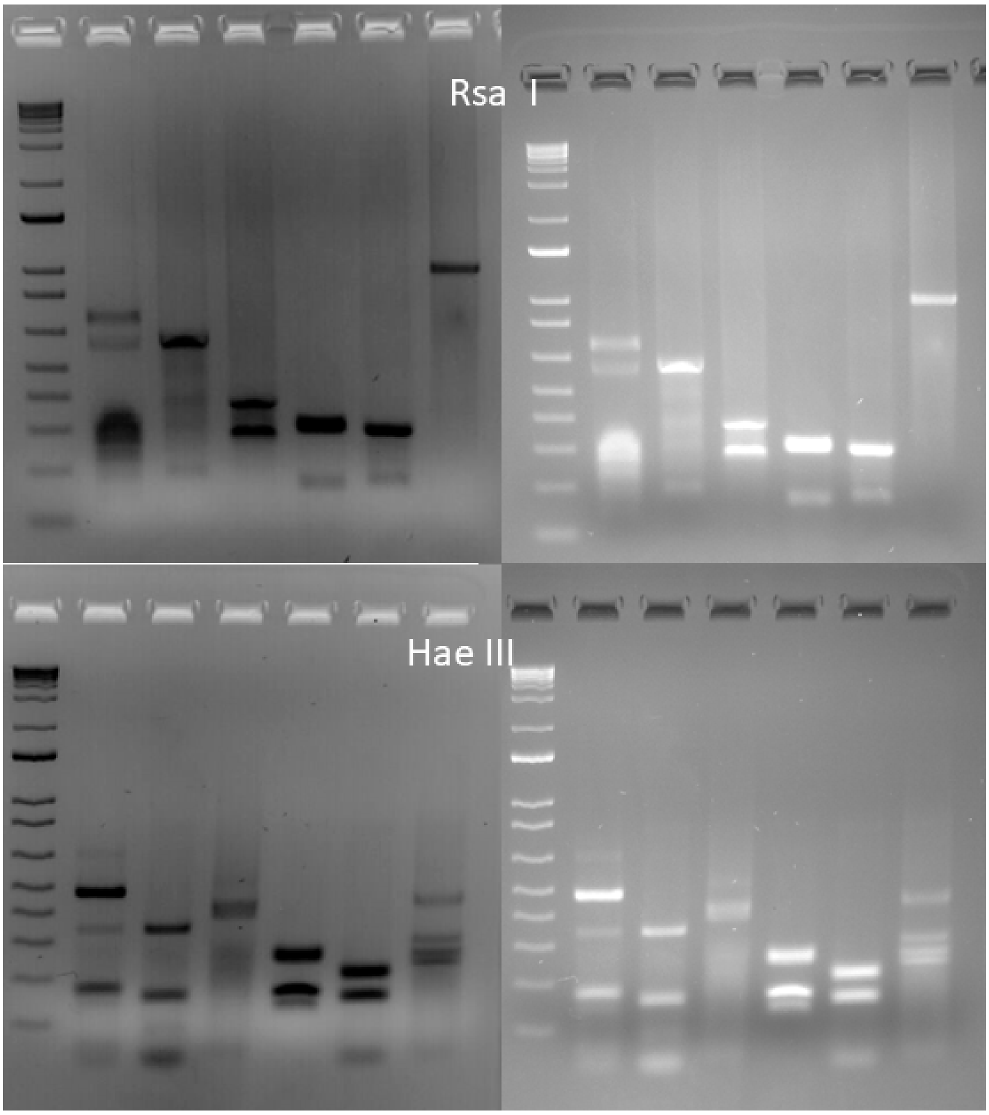

Figure S4: Restriction digestion with *Hae* III enzyme. Experiment conducted to check the reproducibility.

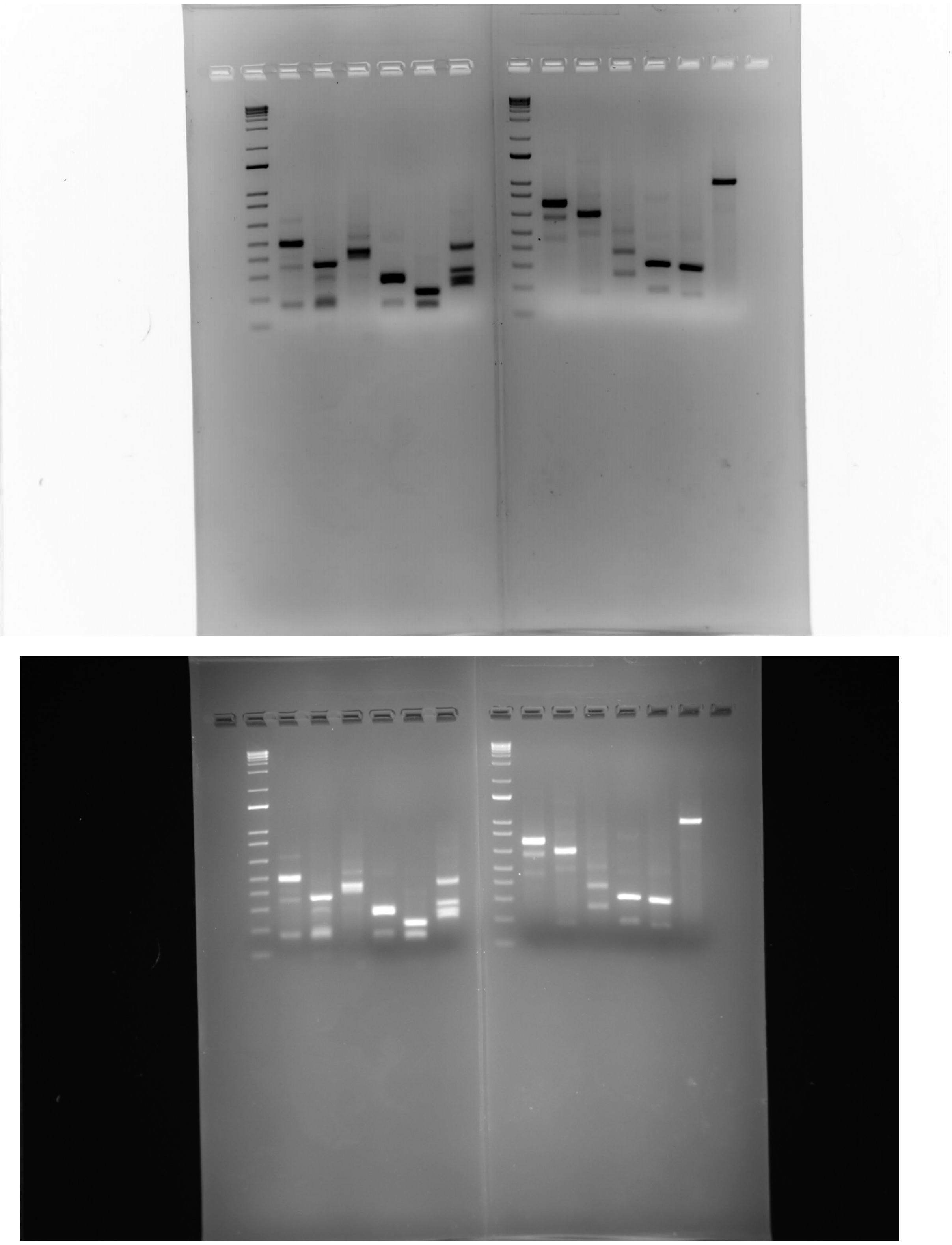

Figure S5: Uncropped and negative gel of restriction digestion *Hae* III and *Rsa* I.

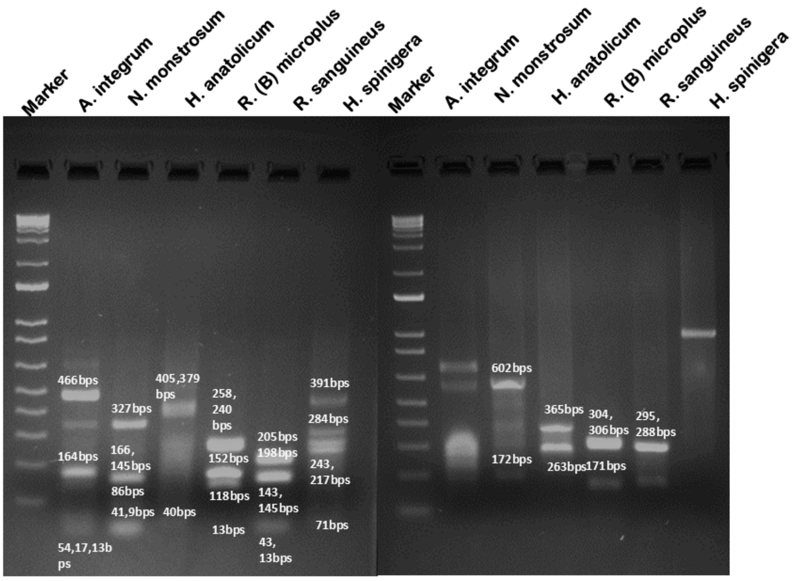

Figure S6: Different band pattern annotations from different tick vectors by using *Hae* III and *Rsa* I restriction enzymes.

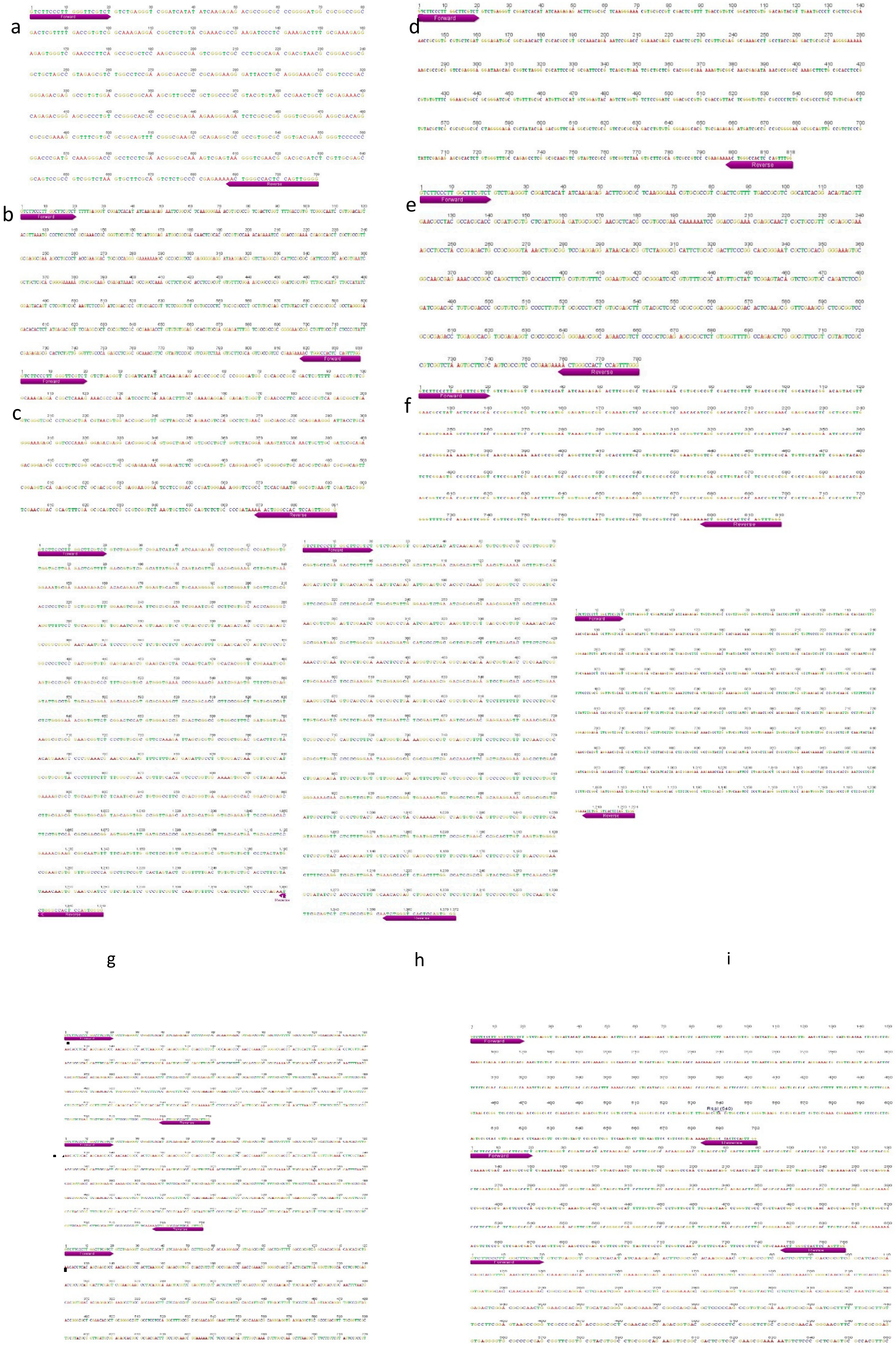

Figure S7: Alignment and primer annotation s of tick vectors from different parts of world (sequences are retrieved from NCBI). All the images are editable.

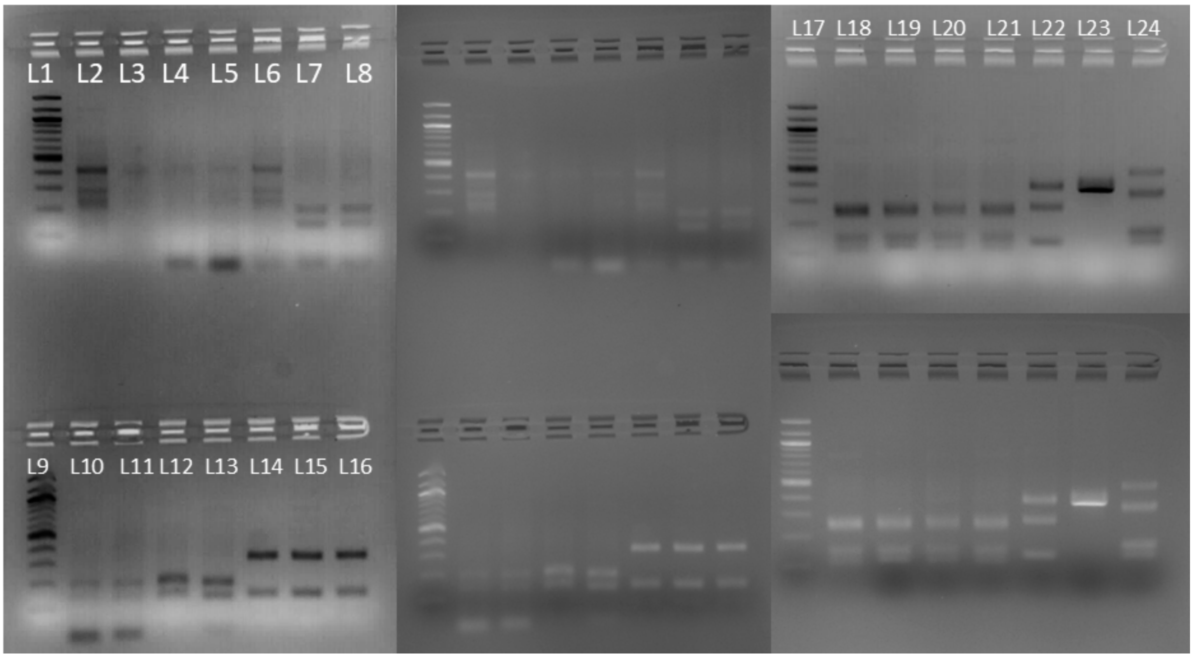

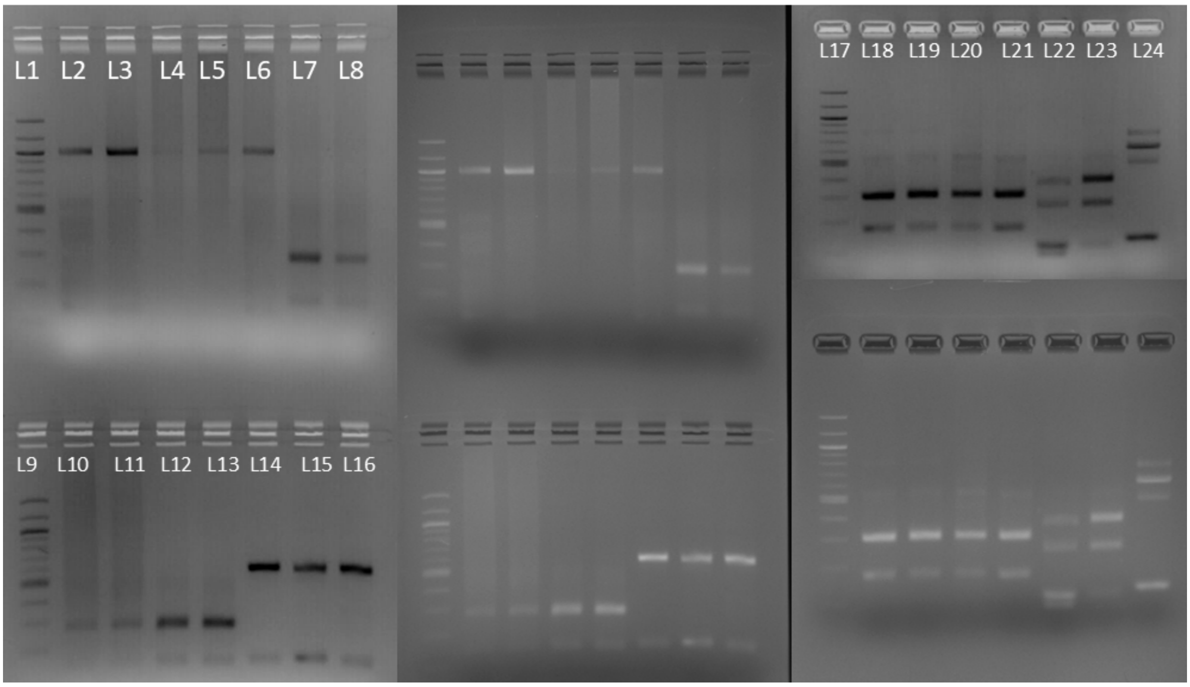

Figure S8: Restriction digestion of ITS-2 fragments amplified from Indian tick vectors. Upper gel figure shows the digestion of ITS-2 fragment with *Hae* III restriction enzyme and lower gel shows the digestion of *Rsa* I restriction enzyme. L1, L9, L17-Marker, L2-L6- *Haemaphysalis spinigera* (nymphs from different sites), L7- L13- *Rhipicephalus sanguineus* (adults from different sites), L14-L16- *Nosomma monstrosum* (adults from different sites), L18-L21- *Boophilus microplus* (adults and nymphs from different sites), L23- *Hyalomma anatolicum* (adult), L24- *Amblyomma integrum* (adult)
